# Retrospective analysis of 514 cases of tibial plateau fractures based on morphology and injury mechanism

**DOI:** 10.1186/s13018-019-1321-8

**Published:** 2019-08-23

**Authors:** Kehan Hua, Xieyuan Jiang, Yejun Zha, Chen Chen, Bosong Zhang, Yujiang Mao

**Affiliations:** Department of Orthopedic Trauma, Beijing Jishuitan Hospital, Fourth Clinical College of Peking University, Beijing, China

**Keywords:** Fracture, Tibial plateau, Classification, Injury mechanism, Morphology, Posterior condylar triad

## Abstract

**Background:**

Tibial plateau fractures remain a clinical challenge due to the complexity of the fracture patterns which have been repeatedly categorized by many researchers. However, limitations do exist in some respects. So we aimed to reclassify tibial plateau fractures based on injury mechanism and morphological characteristics.

**Methods:**

Five hundred fourteen cases of tibial plateau fractures were enrolled. The X-rays and CT scans were analyzed.

**Results:**

According to our observation and analysis, tibial plateau fractures can be categorized into the following six types: (1) Lateral condylar fractures (axial force applied while knee extending in valgus position). Two hundred fifty-one cases were included (48.83%). (2) Fracture dislocation (multiple forces especially rotational stress while knee extending). Fifty-five out of 514 cases belong to this pattern (10.70%). Correction of the subluxation remains primary and crucial during surgical procedures. (3) Simple medial condylar fractures (axial force applied while knee extending in varus position). One third of which were associated with an avulsion fracture of fibular head. Fifteen cases were included (2.92%). (4) Bicondylar fractures (axial forces applied while knee extending). One hundred twelve cases were included (21.79%). Surgical algorithm greatly depends on soft tissue conditions. (5) Posterior condylar fractures (axial stress applied while knee flexing). Sixty-five cases were seen in our study (12.65%), most of which were associated with an avulsion fracture of the intercondylar eminence (49/65, 75.38%). The fracture of posteromedial part, posterolateral part, and intercondylar eminence forms a unique pattern of injury defined as “Posterior Condylar Triad.” (6) Anterior condylar compression fractures (axial, varus, or valgus forces applied while knee overextending). Posterior structural complexes, crucial ligaments, or even popliteal arteries are prone to be damaged. Sixteen cases were identified (3.11%).

**Conclusion:**

Our classification system has instructive significance in overall preoperative evaluation of fracture features and soft tissue problems as well as guiding clinical management for better functional outcomes.

## Background

Tibial plateau fractures are among the most difficult intra-articular fractures to handle and remain a surgical challenge due to the complexity of the fracture patterns [1]. Schatzker et al. [2] categorized tibial plateau fractures into six different types from I to VI based on the site (medial condyle, lateral condyle, and metaphysis), the morphology (split and depression) of the fractures, and the overall prognosis. This classification system is by far the most widely adopted system not only in the literature but also in the clinical practice, as it acts as a guide to appropriate surgical strategy by accurately describing fracture line orientation and morphologic configuration for each pattern [3]. The Hohl and Moore classification [4] emphasized the significance of fracture displacement, sagittal instability, and dislocations based on both anteroposterior and lateral view of X-ray as well as injury mechanism to some extent. AO/Orthopaedic Trauma Association classification is more comprehensive, but it is not universally used due to its complexity and difficulty of memorizing the numerous possible subtypes [5]. Even so, there are still certain fractures that cannot fit into these three traditional systems. Recently, Luo et al. [6] introduced the “three-column concept” based on the transverse view of CT scans, defined as the lateral, medial, and posterior column. This was an important advance in the classification and treatment of tibial plateau fractures as it emphasized the significance of the posterior pillar which filled the vacancy for certain types of fracture configurations that may not conform to the traditional Schatzker classification. Although Luo’s concept distinctly describes the involvement of different columns, the morphology and injury mechanism are not included. In order to improve the classification system for tibial plateau fractures, we retrospectively collected and analyzed X-rays and CT scans of 514 cases, and reclassified the fractures based on morphology and injury mechanism.

## Methods

We retrospectively collected and analyzed the X-rays and 3D CT images of 514 consecutive cases of tibial plateau fracture, treated at our hospital from January 2010 to December 2012. The approval was given by the institutional review board (IRB) and has been performed in accordance with the ethical standards as laid down in the 1964 Declaration of Helsinki and its later amendments or comparable ethical standards. The included cases were assigned a Schatzker classification after review of X-rays based on the consensus of three authors. 3D CT scans were also evaluated. Patient demographics are summarized in Table 1. Furthermore, preoperative imaging data (X-rays and 3D CT scans) of all cases were evaluated, analyzed, and reclassified according to fracture morphology and injury mechanism using certain indicators, including fracture site (medial condyle, lateral condyle, and metaphysis), involvement of intercondylar eminence, tibiofemoral congruence, and morphologic characteristics (split and depression) of the fractures.

**Table 1:** Patient demographics

Total number of patients	512
Sex	
Male	337
Female	175
Average age (range, standard deviation)	45.64 (15–82, ± 12.54)
Fracture classification (Schatzker)	
I	18 (3.50%)
II	261 (50.78%)
III	4 (0.78%)
IV	43 (8.37%)
V	114 (22.18%)
VI	74 (14.40%)

## Results

According to fracture morphology and injury mechanism, tibial plateau fractures can be reclassified into six different types.

### Lateral condylar fracture (valgus type)

**Fig. 1:**
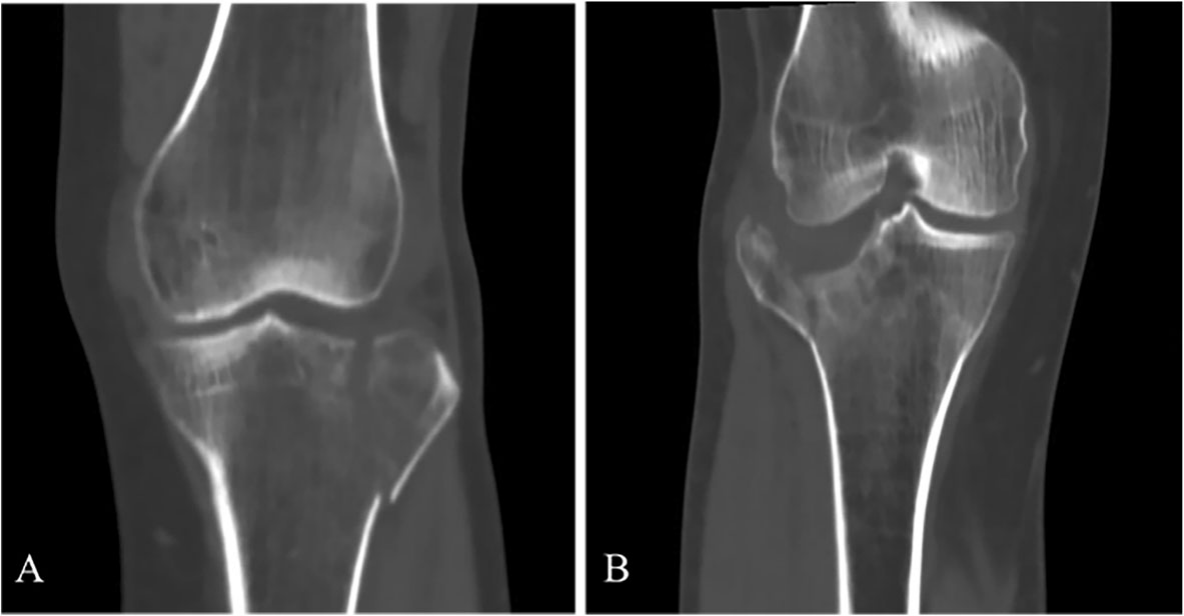
Lateral condylar fracture/valgus type: simple lateral condyle split (Schatzker typeI) (**a**) and lateral depression + split (Schatzker type II) (**b**)

Valgus injury/lateral condylar fracture is the most common pattern in tibial plateau fracture (see Fig. 1). The injury mechanism is defined as axial force applied to the knee extending in valgus position. The morphology of the tibial platform can present as a lateral condylar split and depression of the articular surface, or at times, a combination of both. This type of fracture includes Schatzker I, II, and III excluding posterolateral fractures. Two hundred fifty-one out of 514 fractures (48.83%) fit into this category.

### Fracture dislocation (complex force type)

**Fig. 2:**
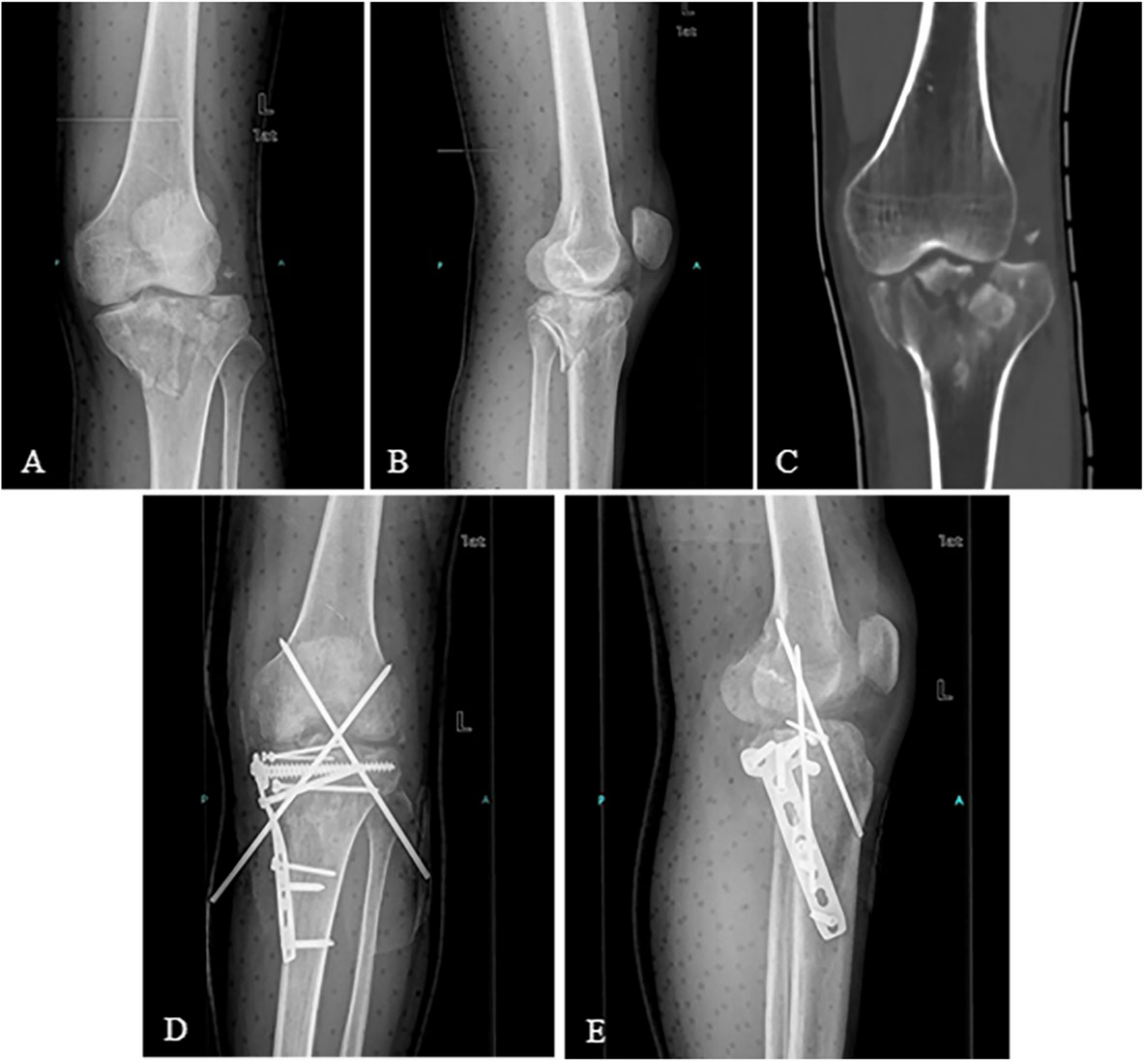
Fracture dislocation type: typical Schatzker type IV, medial condyle matches the femur while the lateral part deviates to rotary subluxation (**a**–**c**). Knee instability after open reduction and internal fixation, so two intersect K-wires were inserted (**d**, **e**)

**Fig. 3:**
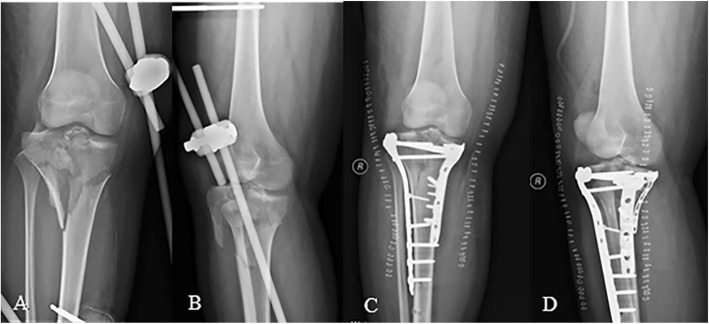
Fracture dislocation type: Schatzker type VI with dislocation treated by external fixator (**a**, **b**). Lateral subluxation still exists after ORIF (**c**, **d**)

This type of injury is comprised of typical Schatzker type IV and type VI with lateral subluxation (55/514, 10.70%) as demonstrated in Figs. 2 and 3. And according to our classification system, Schatzker type IV fractures are segmented into four different subtypes (see Table 2). The typical type IV is characterized by having the medial condyle split off, with fracture lines starting from outside the tibial spine then extending medially and downwards. The medial fragment matches the femur perfectly while the lateral part deviates to rotary subluxation, which results in broadening of the lateral tibial platform shown in the AP view of plain film. It takes complex forces, including valgus, varus, axial, and especially rotational force, to cause lateral subluxation. And as such, this is the most intractable type of injury for surgeons to handle. Even if excellent reduction and fixation have been achieved, subluxation may still exist because of concomitant ligament injury and fracture of the intercondylar eminence, resulting in knee instability and loss of range of motion (ROM).

**Table 2:** Subtypes of Schatzker type IV

Subtypes	Injury mechanism	Cases	Percentage
Simple medial condyle	Varus force, knee extended	15	34.88
Posteromedial split	Varus force, knee flexed	5	11.63
Anteromedial depression	Varus force, knee hyperextended	2	4.65
Fracture dislocation^*^	Rotary and shear forces (mainly)	21	48.84

*Typical type of Schatzker type IV fractures

### Simple medial condylar fracture (varus type)

**Fig. 4:**
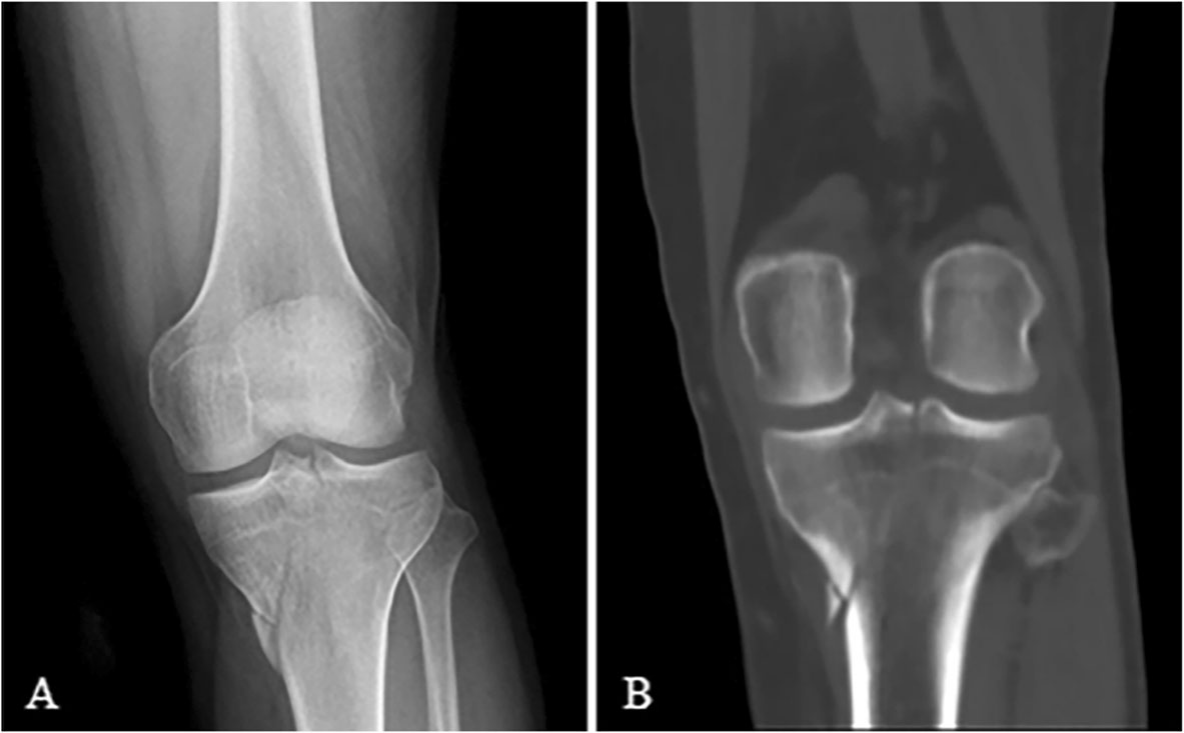
Simple medial condylar fracture/varus type: medial condylar split off or collapse with fracture lines starting from either inside or through the spine of the tibia, extending medially and downwards but with no subluxation (**a**, **b**)

These fractures share similar morphologic configuration with typical Schatzker type IV injury in the absence of subluxation between the tibia and femur. However, the remarkable difference lies in the formation of fracture lines which start from either inside or through the tibial spine (see Fig. 4). This type of injury happens when axial force is applied to the knee extending in varus position. There are 15 out of 514 cases (2.92%) in our study which have such morphologic features and injury mechanism.

### Bicondylar fracture (extension type)

**Fig. 5:**
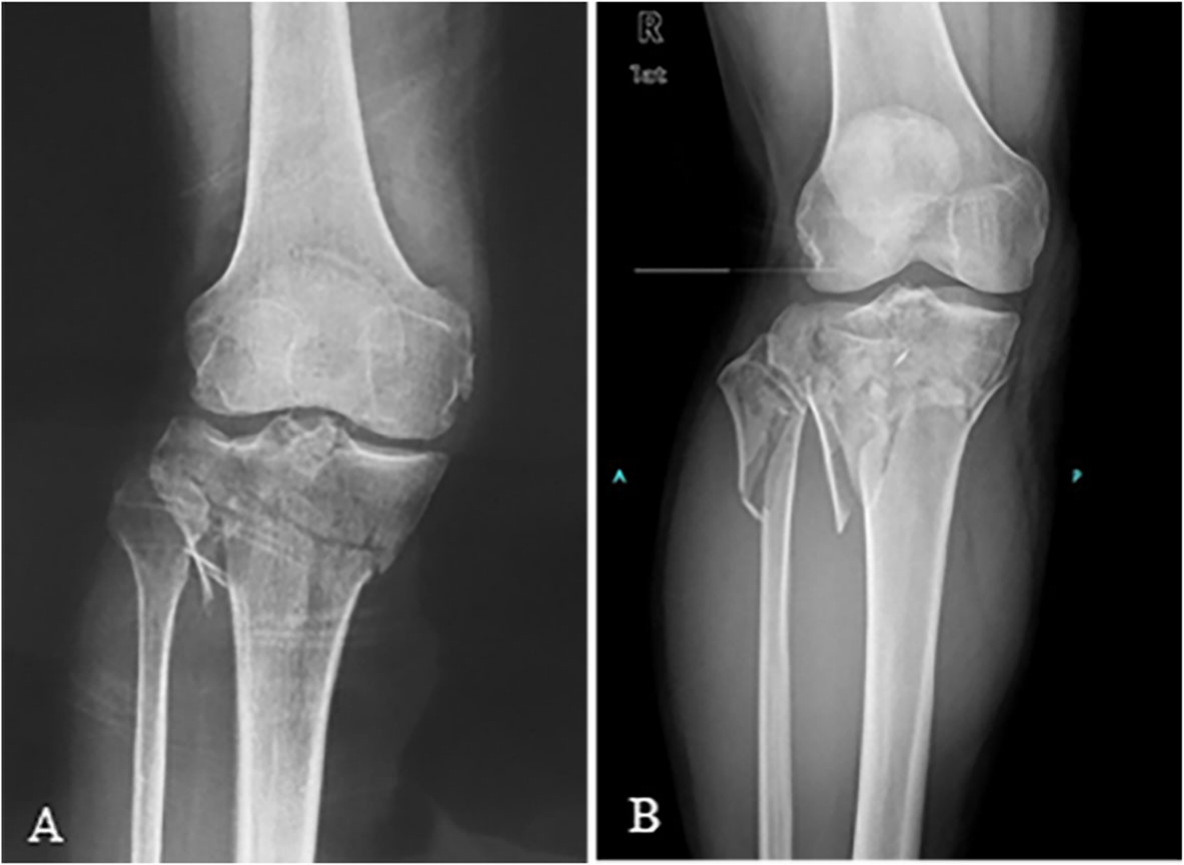
Bicondylar fracture/extension type: split and/or depression of both medial and lateral condyle without subluxation or dislocation (**a**). Metaphyseal dissociation may be involved if the joint is loaded with enough axial forces (**b**)

Fracture morphology of this type of injury is characterized by split and/or depression of both medial and lateral condyles in the absence of dislocation or subluxation of knee joint (see Fig. 5). Schatzker type V as well as type VI with well-integrated tibiofemoral congruence is concluded in this pattern (21.79%, 112/514 cases). The mechanism of the injury is characterized by strong axial force applied to the knee in extension. Meta-diaphyseal dissociation, such as split and comminution, may be involved if the joint is loaded with enough axial stress.

### Posterior condylar fracture (flexion type)

**Fig. 6:**
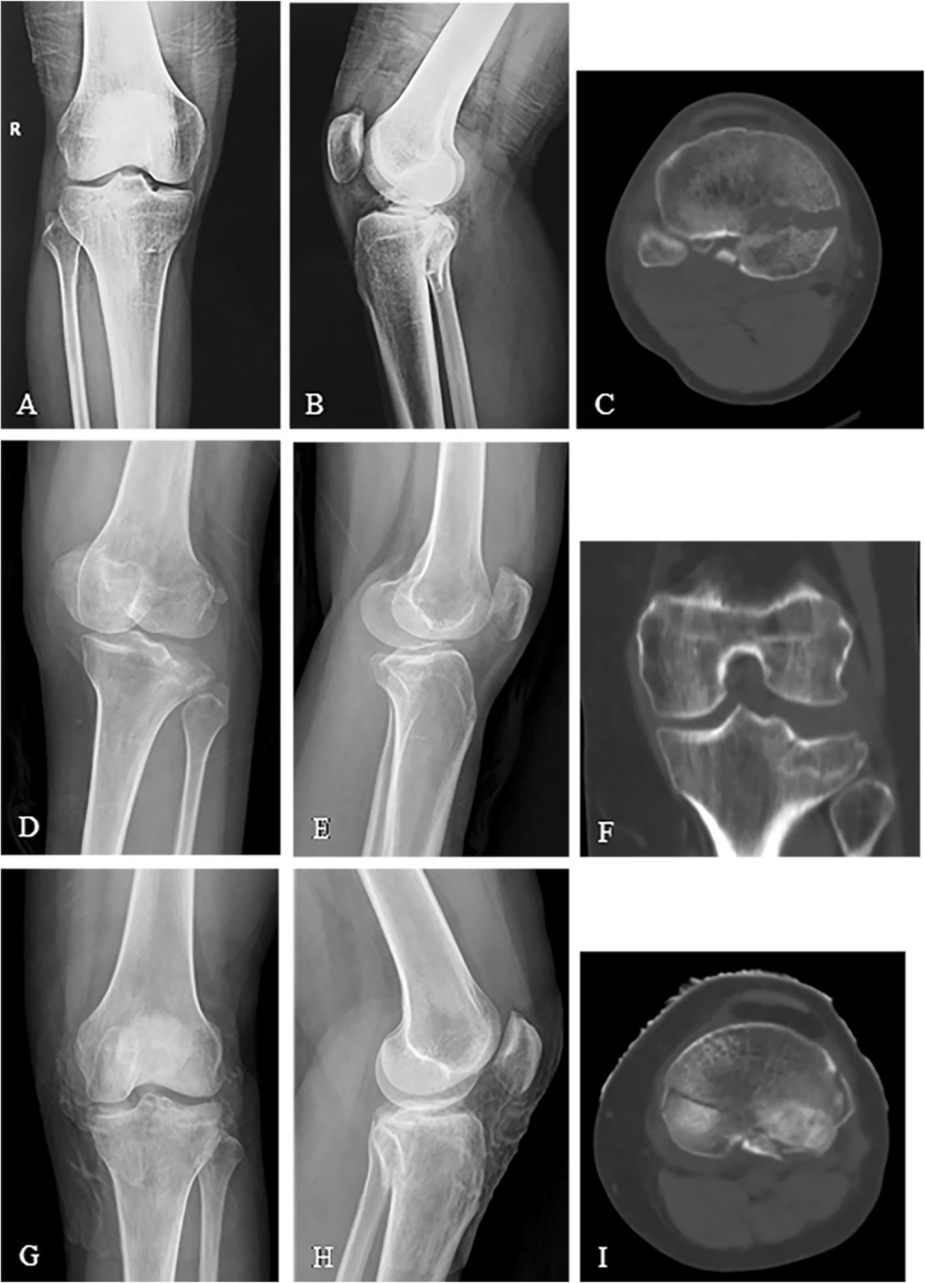
Posterior condylar fracture can be subdivided into three patterns: posteromedial split (**a**–**c**), posterolateral depression (**d**–**f**), and a combination of both (**g**–**i**)

**Fig. 7:**
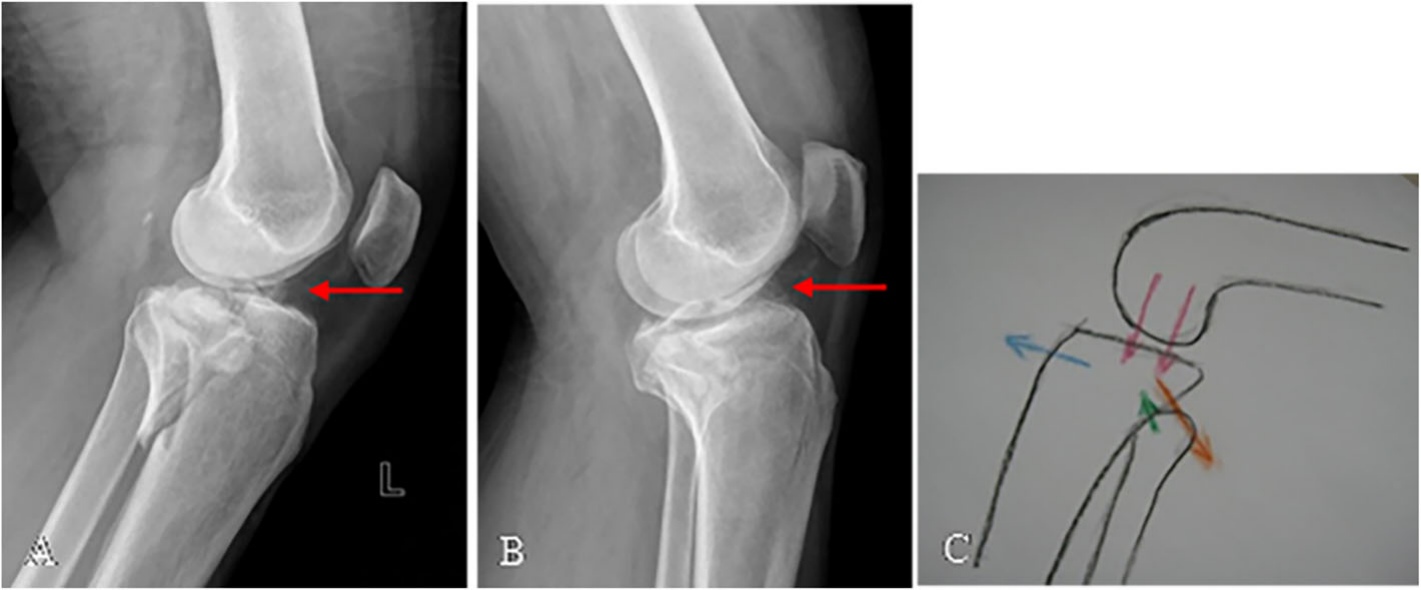
Posterior condylar fracture/flexing type is often associated with intercondylar eminence avulsion fracture (**a**, **b**). Push-pull mechanism (**c**)

Such injury has been overlooked by the Schatzker classification, but it actually is not an uncommon type of tibial plateau fracture with 65 cases (12.65%) seen in our study. According to fracture morphology, posterior condylar fractures can be further divided into three subtypes (see Fig. 6): posteromedial split (7.69%, 5/65), posterolateral depression or with split (49.23%, 32/65), and the combination of both (43.08%, 28/65). It is noteworthy that avulsion fracture of the intercondylar eminence was seen in 49 cases (75.38%) which is a characteristic manifestation for posterior condylar fractures (see Fig. 7). The mechanism of injury is defined as axial, varus, or valgus loading forces applied to the posterior side of the tibial platform with the knee in flexion. The posterolateral condyle would be the first to crumble under such stress owing to its relative frailty in bone quality. Furthermore, the head of the fibula supports this area through the proximal tibiofibular joint, functioning as a buttress plate. Therefore, articular depression tends to occur rather than a split. As the stress ultimately strikes the posteromedial condyle, coronal shear or split is prone to occur instead of compression fracture due to the high density of subchondral bone. With the posterior condyle being injured in flexion position, the anterior cruciate ligament (ACL) is pulled in tension which may lead to rupture or even avulsion fractures (*push-pull mechanism*).

### Anterior condylar compression fracture (hyperextension type)

**Fig. 8:**
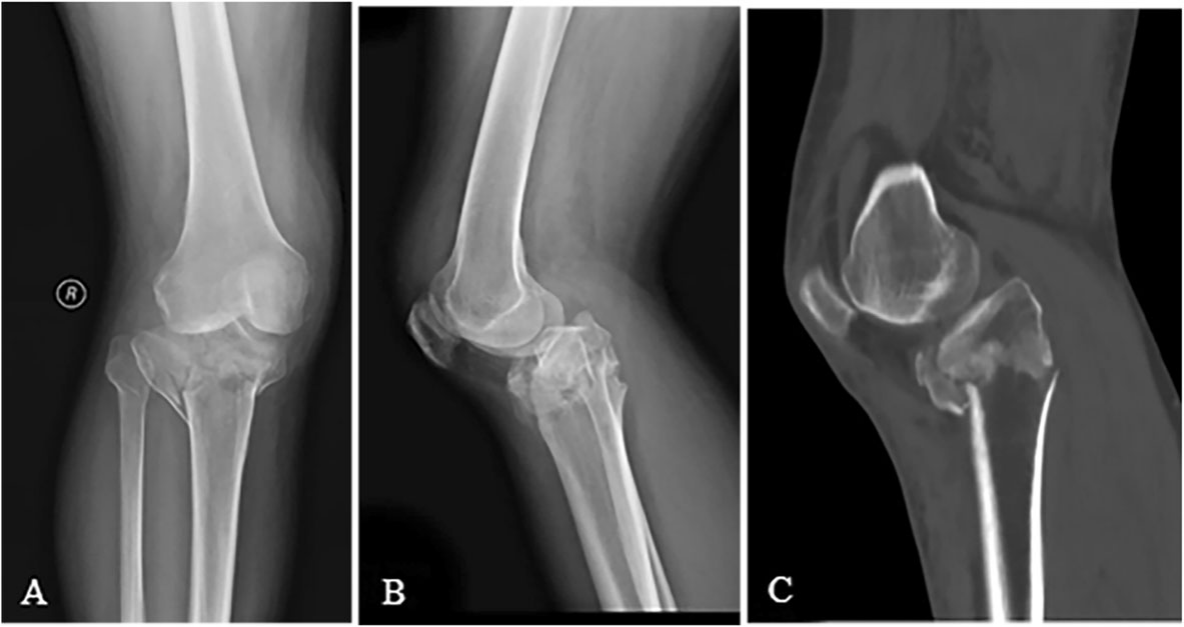
Anterior condylar depression fracture with the posterior condyle intact caused by axial force applied to the knee in an overextended position (**a**–**c**)

This type of fracture is rarely seen in clinical practice (16/514, 3.11%). It is distinguished by notable depression in the anterior part of the tibial condyle while the posterior column is intact (see Fig. 8). The fracture occurs when axial, varus, or valgus compressive forces are applied to the knee in an overextended position (< 0°). On further evaluating the morphology, more than 80% of the cases have bicondylar depression (13 cases) including 9 cases (9/16, 56%) of Schatzker type V and 4 cases (4/16, 25%) of Schatzker type VI fracture. Anteromedial or anterolateral depression described as unicondylar fracture only accounts for 19% (3 cases) of the 16 cases, with 2 cases and 1 case for each pattern, respectively.

## Discussion

### Limitations of the Schatzker classification system

In 1979, Schatzker et al. [2] introduced a simplistic classification system mostly based on an analysis of morphologic fracture seen on an AP X-ray review. However, the system divides tibial plateau fractures into six distinct types with severity increasing and overall prognosis worsening from I to VI. The structure of tibial platform is complicated and subtle, so it may be inaccurate and incomprehensive to evaluate the actual morphology of tibial plateau fractures only based on plain films. Maripuri et al. [7] evaluated 50 tibial plateau fractures and noted that the mean kappa coefficient values for Schatzker classification were 0.47 while the mean percentage of agreement was merely 0.59 for inter-observer reliability. Zhu et al. [8] obtained similar result finding the mean kappa values for inter-observer reliability to be 0.567. Besides, several studies have shown that 2D or 3D computed tomography and MR imaging are more accurate than plain film radiography for the characterization and classification of tibial plateau fractures with excellent inter- and intra-observer reliability [9–11]. After evaluating and analyzing X-rays and CT scans of all cases, we found that the Schatzker classification has the following limitations.

First, posterior condylar tibial plateau fractures, conforming to the morphology of 65 cases in our study, are not included. It is almost impossible to identify and evaluate fractures with posterior condylar involvement by X-rays alone due to the overlap between the fracture and the normal bone. Lack of clinical experiences may also contribute to such fractures being overlooked.

Secondly, Schatzker type IV is defined simply as a medial condylar fracture, but we found that typical Schatzker type IV fractures have the characteristic features of lateral condylar subluxation which is difficult to deal with due to its stubborn persistence even if good reduction and fixation have been accomplished. For this reason, the traditional definition of Schatzker type IV fracture did not adequately described the actual characteristics of fracture morphology, injury mechanism, and overall prognosis. Thus, it is essential for orthopedists to have a deeper and a further understanding of this type of injury.

Thirdly, Schatzker type III is defined as simple lateral condyle depression without any cortical split. After evaluating and summarizing the first 200 cases in chronological order, we were surprised to find that not even 1 case of typical Schatzker type III fracture was seen. Gardner et al. [12] analyzed MRI of 103 cases of tibial plateau fractures with no pure depression injuries detected neither. However, with more data collected and a deeper understanding of CT imaging, we noted that a pure depression fracture dose exists but only accounts for a very small portion (4/514, 0.78%).

### Reclassification based on injury mechanism

#### Lateral condylar fracture (valgus type)

According to Schatzker’s classification, type I, II, and III are unicondylar lateral fractures, defined as pure lateral condylar split, depression combined with split, and pure depression, respectively. Schatzker type II accounts for more than 50% of tibial plateau fractures followed by type I while type III is barely seen in clinical practice. They share the same injury mechanism which is axial force applied to the knee extending in valgus position. Meanwhile, there are no significant differences between them in surgical strategy. Therefore, it is simple and reasonable to merge them into one single pattern, namely “Lateral condylar fracture”. Among such injuries, however, posterolateral condylar fracture should be excluded owing to its distinct peculiarity in injury mechanism and fracture site. Theoretically, if articular step off (≥ 2 mm) and/or condylar widening (> 5 mm) are present, surgical management is preferable. The classic method has been to elevate the articular surface, reduce the condylar split if necessary, and fill the depressed cavity with bone graft via an anterolateral approach, followed with application of a subchondral raft of screws and an anatomic periarticular fixed angle locking plate [2, 3].

#### Fracture dislocation (complex force type)

Similar to the Hohl and Moore classification system, we also lay stress on the significance of fracture dislocation based on injury mechanism and morphological features as well as clinical relevance. We believe that thorough comprehension of fracture-dislocation is of great importance in the management of tibial plateau fractures. Obviously, it takes multiple stresses especially rotary forces and much more energy to cause fractures with tibiofemoral dislocation or subluxation, which means severe soft tissue damage along with ligamentous rupture, meniscal injuries, and even compartment syndrome. It is worth noting that the fracture lines of such pattern originate from merely outside the tibial spine, so ACL and PCL (posterior cruciate ligament) cannot stabilize the lateral part which leads to the typical lateral subluxation. Given the extensive injury, the surgical management needed to deal with such damage in order to optimize postoperative outcome is complicated and varies with each individual. In general, such serious injuries are preferably approached in a staged manner respecting the principle of damage control, referring to the application of temporary joint-bridging external fixators [3, 13], meanwhile delaying the definitive internal fixation until such time as physiological status and soft tissue condition have improved. Because of the concomitant injuries, however, subluxation or knee instability may still exist even after reduction and internal fixation have been carried out in the best scenario. Therefore, when it comes to definitive fixation, the lateral subluxation must be dealt with in priority. Closed reduction inverse to the injury mechanism by traction and rotation (often externally) remains the initial and indispensable management. But, in certain cases, open reduction is needed via an additional lateral incision, and it turns out that the lateral meniscus is stuck in the gaps between medial and lateral fragments.

#### Simple medial condylar fracture (varus type)

As a subtype of Schatzker type IV fracture illustrated in Table 2, simple medial condylar fracture, caused by varus force applied to the extended knee, is defined as a depression and/or split of the medial condyle without tibiofemoral dislocation or subluxation, accounting for 15 cases in our study (2.92%). Unlike the complex force type, the fracture lines of varus type start from either inside or through the tibial spine without compromising the stability of the lateral column, so articulation is fine. In that case, with good reduction and internal fixation, simple medial condylar fractures tend to have better outcomes in joint stability and functional recovery owing to the relative integrity of the soft tissue envelope and ligamentous structures compared to fractures with articular incongruity. In addition, avulsion fractures of fibular head was seen in one third of pure medial condylar fractures while being relatively rare in other patterns (see Table 3). Similar to the “push-pull mechanism,” the medial condyle of tibia and femur are “pushed” against each other under varus forces while the fibular head carried by the lateral collateral ligament and biceps femoris muscle is “pulled” apart from fibula. We address the significance of stabilizing fibular head avulsion fractures using screws, tension band, suture anchor, or even plates on account of the potential risk of compromising knee stability.

**Table 3:** Distribution of fibular head avulsion fracture

Patterns	Cases	Percentage*
Valgus type	1	1/251, 0.40%
Complex force type	4	4/55, 7.27%
Extension type	5	5/112, 4.46%
Flexion type	0	0/65, 0.00%
Hyperextension type	1	1/16, 6.25%
Varus type	5	5/15, 33.33%
Total	16	16/514, 1.17%

*The percentage of fibular head avulsion fractures found in each pattern and in all cases

#### Bicondylar fracture (extension type)

Schatzker type V and type VI without subluxation compose this pattern which is caused by a strong axial force applied to the knee in extension. Although controversies do exist on distinguishing Schatzker type V from type VI because sometimes it is difficult to recognize the dissociation of metaphysis and diaphysis, they do not have many differences in the essentials of surgical strategy and postoperative rehabilitation. For that matter, it is logical to put them together as one single type of injury resembling the “Bicondylar type” in Hohl and Moore classification system. Generally, all bicondylar fractures need operative intervention if the soft tissue condition permits [14]. Such type of fractures tends to have different levels of soft tissue problems which are decisive factors for planning surgical algorithm. The traditional operative strategy is to approach the medial and lateral condyles via anterolateral and posteromedial incisions, respectively, then restore articular congruity as well as normal mechanical alignment using dual fixed angle plates. Sometimes, however, single lateral plate may also do the trick depending on the fracture lines and the severity of the injuries. What is more, it is of great necessity to protect the soft tissue envelope from devitalization by employing minimal surgical procedures and protective perioperative care.

#### Posterior condylar fracture (flexion type)

Posterior tibial plateau fractures (PTPFs) are not uncommon in tibial plateau fractures but relatively rare compared to the other patterns. PTPFs have been neither systematically nor sufficiently demonstrated by the Schatzker classification because CT scanning was not widely adopted back then [2]. Chen et al. [15] proposed a detailed classification of PTPF and divided it into (I) posteromedial split, (II) posterolateral split, (III) posterolateral depression, (IV) posterolateral split with depression, and (V) posteromedial split combined with posterolateral depression. We found an interesting fact that almost all Chen et al.’s type V fractures are coupled with avulsion fractures of the intercondylar eminence (26/28, 92.86%), forming a unique morphological triad which can be explained by the “push-pull mechanism” illustrated above. For this reason, we address them as the “Posterior Condylar Triad”, emphasizing the necessity of recognizing an avulsion fracture of the intercondylar eminence preoperatively if a PTPF (especially posteromedial split plus posterolateral depression) has been identified, owing to its essential role in stabilizing the knee joint for early postoperative rehabilitation. Thus, more attention should be paid to the coronal view of the CT scan looking for avulsion fractures or to MRI for further examination for ligament tear or rupture.

Due to the complexity of injury patterns including split and/or compression fracture of the posteromedial and posterolateral tibial platform as well as the avulsion fracture the intercondylar eminence, neither single one of the subtypes in the Hohl and Moore classification system, such as the “Split type,” can fully describe the particular fracture characteristics independently. Therefore, the analysis and evaluation based on the combination of injury mechanism (*push-pull mechanism*) and morphological features (*Posterior Condylar Triad*) are more accurate and comprehensive to demonstrate this specific subtype in our study.

As for surgical treatment, a posterior approach is widely adopted as it allows satisfactory visualization of the posterior aspect, thus reducing the risk of injuring adjacent blood vessels and nerves [16–18]. He et al. [19] introduced a posterior L-shaped approach for the treatment of bicondylar PTPFs and came to a conclusion that this approach is safe as well as effective, and it does not require osteotomy, tendotomy, or division of muscles while obtaining sufficient surgical field. In our study, we tend to use two parallel incisions, posterolateral and posteromedial, to expose the posterior condyle. An extra anteromedial incision may be needed if the intercondylar eminence is involved.

#### Anterior condylar compression fracture (hyperextension type)

If varus, valgus, or axial forces are applied while knee hyperextending, anteromedial, anterolateral, or anterior bicondylar depression will occur, respectively. The typical manifestation found on X-rays is a reversed posterior slope. According to the “diagonal injury mechanism” [20] (similar to our “push-pull mechanism”), such fractures are more likely to combine injuries of the posterolateral complex and/or posteromedial ligaments, even with fibular head fractures, ligamentous avulsion fractures, and posterior metaphyseal cortical tension rupture. More severely, popliteal artery may be injured (contusion or rupture), but it can be easily misdiagnosed because it is often hard to tell it from compartment syndrome. And peripheral blood supply, such as arteria dorsalis pedis and posterior tibial artery, is sometimes unreliable due to collateral circulation. For that matter, arteriography should be conducted even with slightly suspicion of artery injury. Conesa et al. [21] reported a case of anteromedial tibial plateau fracture associated with posterolateral complex injury diagnosed by MRI, and the lateral collateral ligament as well as biceps tendon was repaired and supplemented with a peroneal tendon allograft which emphasizes the importance of MRI and repair of posterior structural damage. Unfortunately, many patients in our study did not have MRI checked preoperatively mainly due to overbooking and the absence of MRI machine in the emergency room. And of course, lack of experience and knowledge is also a possible reason for incomplete preoperative diagnosis. Gonzalez et al. [22] compared HEBTP (hyperextension bicondylar tibial plateau fracture) patients (15 cases) with non-HEBTP patients (69 cases) and found that HEBTP patients have higher Short Musculoskeletal Function Assessments (SMFA) and pain scores, indicating worsen functional outcomes and a tendency of having associated soft tissue damage and developing posttraumatic osteoarthritis. Since hypertension tibial plateau fractures remain a huge clinical challenge for orthopedic surgeons and no consensus has been reached on the treatment of these fractures, more evidence-based clinical trials are needed in the future. Therefore, in our department, we tend to use spanning external fixators as early stage management due to the difficulties of dealing severe overdepressed fragments using internal fixation techniques.

Our research does have certain limitations. First, our study is retrospective so the injury mechanism is based on our analysis of fracture morphological characteristics and clinical experiences over hundreds of tibial plateau fractures. But we do have noticed that many patients cannot recall the exact injury mechanism or even have a false memory due to coma, anxiety, etc. So medical history collecting or telephone follow-ups may be unreliable. Secondly, comprehensive follow-up results are needed to better distinguish each pattern from the aspect of postoperative functional prognosis. Thirdly, not all of our patients had MRI checked before surgery so there may be some missed diagnosis of undisplaced fractures or ligament lesions, which means we probably have underestimated or misdiagnosed some severe injuries. Therefore, comprehensive follow-ups and further clinical trials need to be conducted to improve our classification system.

## Conclusion

Based on injury mechanism and fracture morphology of 514 tibial plateau fractures, we classified them into six different types, which are valgus type, complex force type, extension type, flexion type, hyperextension type, and varus type. Our classification system is meant to help orthopedic surgeons better understand fracture characteristics so as to guide appropriate surgical treatment and estimate clinical outcomes preliminarily. More importantly, it serves as a reminder for doctors to pay close attention to collateral injuries, such as avulsion fractures of the intercondylar eminence and fibular head as well as damages to important ligaments or structural complexes, which are crucial for knee stability and articular congruity. Therefore, our classification system has instructive significance in the overall preoperative evaluation of fracture features and soft tissue problems as well as guiding clinical management for better functional outcomes.

## Data Availability

The datasets generated and/or analyzed during the current study are not publicly available because another study of our institution requires the datasets in this article but are available from the corresponding author on reasonable request.

## Abbreviations

ACL: Anterior cruciate ligament
HEBTP: Hyperextension bicondylar tibial plateau fracture
IRB: Institutional review board
PCL: Posterior cruciate ligament
PTPF: Posterior tibial plateau fracture
ROM: Range of motion
SFMA: Short Musculoskeletal Function Assessments
